# Sensors: Views of Staff of a Disability Service Organization

**DOI:** 10.3390/jpm3010023

**Published:** 2013-02-22

**Authors:** Gregor Wolbring, Verlyn Leopatra

**Affiliations:** 1 Department Community Health Sciences, Faculty of Medicine, Stream of Community Rehabilitation and Disability Studies, University of Calgary, 3330 Hospital Drive NW, Calgary, Alberta, T2N 4N1, Canada; 2 Bachelor of Health Sciences, Faculty of Medicine, University of Calgary 3330 Hospital Drive NW, Calgary, Alberta, T2N 4N1, Canada; E-Mail: vgsleopa@ucalgary.ca

**Keywords:** sensor, disabled people, personalized medicine, participatory medicine, quantified-self, disability service organization

## Abstract

Sensors have become ubiquitous in their reach and scope of application. They are a technological cornerstone for various modes of health surveillance and participatory medicine—such as quantifying oneself; they are also employed to track people with certain as impairments perceived ability differences. This paper presents quantitative and qualitative data of an exploratory, non-generalizable study into the perceptions, attitudes and concerns of staff of a disability service organization, that mostly serve people with intellectual disabilities, towards the use of various types of sensor technologies that might be used by and with their clients. In addition, perspectives of various types of privacy issues linked to sensors, as well data regarding the concept of quantified self were obtained. Our results highlight the need to involve disabled people and their support networks in sensor and quantified-self discourses, in order to prevent undue disadvantages.

## 1. Introduction

Various types of sensors exist that can be classified in respect to their positioning towards the human body: sensors can be implanted into the body [1,2,3,4,5] and used in various body parts such as in hips [6], they can be externally attached to bodies or be in the vicinity of bodies (wearable sensors) [7,8,9,10,11,12,13] and they can be positioned in the environment such as walls and floors of a home [14,15,16,17,18]. Sensors are used for various purposes in regards to disabled people, including but not limited to health monitoring [19], evacuation and rescue information [19], indoor navigation aid [20], smart home systems that aid disabled people to carry out daily activities in the safety and comfort of their homes [21], real time tracking of disabled people [22], sensor pillow systems [23], assistive living [24], home medical assistance [25], rehabilitation [8], physiological monitoring [11], as health managing system [26] and for mobile health [9]. 

Some of the social and ethical sequelae associated with increased sensor integration into health and personal technologies have been investigated. Fensli, for example, has developed a sensor acceptance model [27,28]. In addition, a human-centered approach to the design and evaluation of wearable sensors has been identified by Totter [7]. Bergman on the other hand, looked into what patients and clinicians want from body-worn sensors [29]. Steele *et al.* identified “sixteen concepts in relation to the elderly participant’s perception, concerns and attitudes towards wireless sensor network systems” [14]. Lubrin *et al.* applied the Unified Theory of Acceptance and Use of Technology (UTAUT) [30,31,32,33,34] model to determine the acceptance of wireless sensor networks in medical institutions and patients’ homes [35]. Some studies looked at caregivers’ views of pervasive computing on people with autism [36], of use of a real time continuous glucose monitoring system in children and young adults on insulin pump therapy [37].

We provide here the results of an exploratory study into the perceptions, attitudes and concerns of staff of a disability service organization towards the use of various types of sensor technologies for their clients. 

Advances in sensor technologies outside and within healthcare applications are seen to have significant impact on privacy [38,39,40,41]. Various papers highlight that all OECD Privacy principles are in high to very high danger of being violated [42,43]. Townsend looked at the trade-off between autonomy and privacy [44]. 

Canada has one of the most comprehensive privacy legislative and policy frameworks in the world [45]. Canadians are protected by two federal privacy laws of general application, the *Privacy Act* and the *Personal Information Protection and Electronics Documents Act* (*PIPEDA*) [45]. To the Privacy Commissioner of Canada, privacy means:

“...the right to control access to one’s person and information about one’s self. The right to privacy means that individuals get to decide what and how much information to give up, to whom it is given, and for what uses [46].

Given the importance of privacy to Canadians and the privacy impact of sensors we present here the views of staff of a Canadian disability service organization on their own privacy, as well as the privacy of their client in general and related to sensor applications. 

People are increasingly seeking personalized health information, with health clients assuming the driver’s seat in respect to both decision making and their health interventions [47]; hence, the concepts of patient driven healthcare and of people driven health research have gained in popularity. We see movements towards a ‘quantified self’ (where people diagnose themselves), patient-driven healthcare and research models [48,49,50,51], and health social networks and participatory medicine with an active health technology market that makes consumer personalized medicine [52,53,54] possible. This shift in the nature of the health client from a passive recipient to an active shaper, and the framing of patients and clients as consumers has broad implications for disabled people and their support environment. We present here the views of the staff of a disability service organization toward the issue of quantified-self. 

## 2. Results

### 2.1. Demographics

Of a total sample of 44 people, 20.5% (n = 9) identified as male and 79.5% (n = 35) as female. As to age, 25.0% (n = 11) were between the age of 18–30; 70.5% (n = 31) were between the age of 30–65 and 4.5% (n = 2) were over 65 years of age. As to their self-understanding of body ability, 93.3% (n = 41) perceived themselves as ‘Normal’ and felt they are perceived by others as Normal. 2.3% (n = 1) saw themselves as ‘Normal’ but felt they are perceived by others as impaired and saw themselves as impaired but felt they were perceived by others as Normal. 47.5% (n = 19) worked in the field more than 8 years; 7.5% worked 5–6 years; 1–2 years and 8month-1 year in the field. Smaller percentages were in between year wise. The majority of respondents that indicated their education level stated it as completed grade 12. The majority of the respondents were care providers—however some identified as program coordinators, program activity staff and admin staff. 

### 2.2. Awareness and Perception towards Different Types of Sensors

To gain an idea of participant awareness and perceptions of various sensor applications and what they thought their client might think, we asked: “Question 16. For the following list of sensors, please select for both yourself and your perceptions of your client whether you have heard of them (are aware they exist) and if you/or your client would use them if they were readily available.”

We chose different sensor systems based on a preliminary literature review that looked at technologies available and in use today. Table 1, Table 2 highlight that (a) there was a difference in awareness for the different types of sensors whereby in each case the staff rated their own awareness as higher than the awareness of their client; (b) staff rated the willingness of using the different sensors higher for themselves than for their client; (c) staff rated for themselves and for their client the willingness to use sensors that are seen to provide information on mental health issues and stress much lower than sensors that were linked to drug delivery and non-mental health information generating devices. 

**Table 1:** Participant awareness and perceptions of various sensor applications for the caregiver.

Myself	I am not aware of this sensor and don’t use/ would not use it	I am aware of this sensor and currently use/would use it	I am aware of this sensor and don’t use/would not use it	I am not aware of this sensor but would use it	ResponseCount
Neurotransmitter sensor; application: to detect various mental health issues	**41.7% (15)**	13.9% (5)	22.2% (8)	22.2% (8)	36
Galvanic Skin Sensor; application: measures stress levels through electrical conductivity of skin	**52.8% (19)**	2.8% (1)	8.3% (3)	36.1% (13)	36
Automatic drug delivery systems; application: for example insulin pumps and pain medications	11.4% (4)	**65.7% (23)**	14.3% (5)	8.6% (3)	35
Wireless wearable health statistic generating devices, example. heart rate watches, Jawbone Up Bracelet; application: to monitor and track common biostats such as blood pressure, heart rate, sleep cycles, nutrients, biomarkers *etc*.	11.4% (4)	**60.0% (21)**	5.7% (2)	22.9% (8)	35

**Table 2:** Participant awareness and perceptions of various sensor applications for their clients.

My Clients	I do not think my client is aware of this sensor and don’t use/ would not use it	I do think my client is aware of this sensor and currently use/would use it	I do not think my client is aware of this sensor and don’t use/would not use it	I do not think my client is aware of this sensor but would use it	Response Count
Neurotransmitter sensors; application: to detect various mental health issues	**58.8% (20)**	0.0% (0)	14.7% (5)	26.5% (9)	34
Galvanic Skin Sensor; application: measures stress levels through electrical conductivity of skin	**52.9% (18)**	2.9% (1)	17.6% (6)	26.5% (9)	34
Automatic drug delivery systems; application: for example insulin pumps and pain medications	**36.4% (12)**	21.2% (7)	12.1% (4)	30.3% (10)	33
Wireless wearable health statistic generating devices, example. heart rate watches, Jawbone Up Bracelet; application: to monitor and track common biostats such as blood pressure, heart rate, sleep cycles, nutrients, biomarkers *etc*.	24.2% (8)	30.3% (10)	12.1% (4)	**33.3% (11)**	33

Of the n = 28 respondents who gave further comments n = 26 could see themselves using such devices based on need, invasion of privacy, utility, accuracy and reliability. To quote one, 

“I think that sensors can provide helpful information to both the user and the people who are assisting the user. I think it needs to be up to the individual user if they want to use the sensor. I also think that depending on the severity of the health issue people would be more or less open to using the sensor.”

Reversibility was seen as important by one,

“I believe that people would be more comfortable if the use of the sensor was for limited amount of time-to assess current situation and to evaluate interventions.”

n = 6 respondents thinking about their clients felt that the use of the applications would be difficult due to cognitive barriers. 

To quote two,

“For myself I currently have no need for any of these devices but if in the future I needed one I would consider using them if they could improve my quality of life. However the clients that I work with are not mentally able to use most of the devices that you mentioned.” 

“Although I have not needed to use any of the above mentioned sensors I am aware of them. For myself I would not hesitate to use or have these devices used on myself. However the participants in our program would be afraid of them and would likely have to have some level of sedation to be able to have many of them used on them and therefore would likely cause some questionable results.”

### 2.3. Benefits and Concerns of Sensors for Health Service and Delivery

To gain an idea of the benefits envisioned we asked, “Q 17 What do you see as the biggest benefits of continued development and use of sensors into health services and delivery?” 

The benefits mentioned by the n = 33 respondents were, (1) improved delivery and safety; (2) ability for individuals to have more control over their own health; (3) sensors can detect what one might not see or know; (4) advancements to monitor various things and help provide support that is needed and consistency; (5) added health information to provide better care; (6) can help ease the work load or can help to discover things that a service worker missed; (7) could save lives and improve quality of health care and treatment. The sensors may enable people to live more independently; (8) to alert caregivers in emergency situations and to more accurately dispense medication. 

To give one quote, 

“The largest benefit would be the provision of accurate medical information that could be provided to medical professionals. This information could result in more accurate medication prescription and quicker diagnosis of underlying health conditions. Fall sensors would also provide for greater safety especially as participants age in homes that are only funded for one sleeping night staff person.”

One made a distinction based on where the sensor is situated, 

“On the body, as it wouldn’t be guaranteed if placing a sensor inside the body would cause further damage”. 

Some (n = 2) felt there would be no benefits. Furthermore, some felt as mentioned before that the uptake might be problematic for their clients. To give one quote,

“All of the above mentioned devices are wonderful tools. However, I feel that they wouldn’t benefit our participants at this time. Perhaps if they had them at a much younger age they would have benefited from them but as most of the participants that I am currently working with are nearing the senior generation I feel that these devices would be more of a hindrance than benefit. One also has to take into consideration the cost of these devices. Most of our participants don't have access to that kind of funding.”

To gain an idea of the concerns envisioned we asked, “Q. 18 What do you see as the biggest concerns of continued development and use of sensors into health services and delivery?”

The 31 respondents that answered the question raised the following concerns; cost (n = 4), devices can fail, invasion of privacy (n = 6,) victimization, could be used without a person' s knowledge and/or consent, job loss, sensors can give false information (n = 5), fear of failing device (n = 3), decrease in human interaction (n = 3), lack of trust in technology, getting away from hands on doctoring and removing the relationship with health provider, blaming people if they choose not to use the sensors,

To use a longer quote,

“The use of sensors can place too much emphasis on the medical model of disability. In this model participants are treated primarily as patients and all behavioral-psycho-social issues tend to be dealt with from a medical perspective. In short, people are treated as though they are sick when in fact they are simply living with a disability. The presence of sensors can also impact the way that non-medically trained staff view themselves. With the accessibility of questionable medical information through the internet and television programs we run a constant risk of staff overestimating their medical knowledge and implementing treatment programs based on this mistake.”

### 2.4. Future role of Sensors in Healthcare Development and Delivery

Many sensor applications are only emerging therefore we asked staff about the future role of sensors in question 19, “Do you foresee sensors as being an important component of future health care development and delivery? Why or why not?”

Of the n = 33 respondents n = 29 felt that sensors will be an important component of future health care development and delivery.

To give some quotes, 

“Certainly there are many who would benefit from these devices. But the majority of them need to have access to them at a young age so they can learn and grow with them. The funding has to be made easier and accessible to all.” 

“I foresee sensors taking over health care. People are always wanting to continue to grow and create things to make life more convenient.” 

“Yes they absolutely could be an important component. Health care is stressed, understaffed and underfunded, this may help with the burden.”

“Yes, as budgets continue to be cut it seems likely that agencies and governments will actively seek out less labor intensive modes of monitoring basic medical information. Also, as technology advances it tends to become more affordable.”

“It could really go either way depending if they have any bugs with the systems and how to know how accurate they would really be in some matters.”

“Yes. - more economical - efficient data collection - enables more accurate information for assessment and treatment - more timely interventions.”

“It would be some assistance in future care as long as we don’t forget the participants that we work with are of human material with individual thinking and feelings. That not every participant is the same.” 

### 2.5. Awareness and Perception towards Quantified Self Movement

The quantified self movement is an emerging phenomenon [48,49,50,51,55]. Therefore, we asked staff in question 20 about their awareness of this phenomenon. Of the 33 staff that answered the question, 97.1% (n = 33) had never heard of this term whereas 2.9% (n = 1) had heard of the term before. 

In question 21 we asked, “Have you been involved at all with the quantified self activities and to what degree? For example: minimally involved—tracking daily food intake, heart rate on runs using a heart rate monitor, *etc*. heavily involved-storing self-generated data online and using to actively monitor self-health and/or pooling data with others. 60.6% (n = 20) of the n = 33 respondents never used a self-tracking device whether they felt it was not needed or they simply were not aware of the technologies whereas 6.1% (n = 2) stated they were very involved, none answered frequently involved; n = 6 or 18.2% occasionally involved; none rarely involved and 15.2% (n = 5) very rarely involved. The ones mentioned as being used by the respondents were calorie intake monitor and heart rate monitor.

We asked further in question 22, “Would you be interested in learning more about and/or taking part in this movement? Why or why not?” Of the n = 33 respondents n = 14 felt no need to learn more whereas n = 15 wanted to learn more with the reason being mostly that it helps one staying healthy.

### 2.6. The Issue of Privacy

Privacy is an important issue debated extensively in the health care and within the sensor application arena. Therefore, we asked various questions in regards to privacy. In question 12 we asked, “Please rate how important each of these forms of privacy are to you (Table 3), as well as which forms of privacy you perceive as being important for your clients” (Table 4). We offered in Question 12 four different types of privacy we felt are impacted by existing or emerging science and technology products (although we did not gave to the respondents the science and technology product that might interfere with the privacy), namely privacy of location (tracking devices), privacy of health information (hacking of e-records); privacy of thought (interference with to come brain machine interfaces), privacy of memory (interference with the artificial hippocampus) and privacy of non-medical information (hacking of banking information…).

In general, respondents ranked privacy as very important for themselves (Table 3) and their clients (Table 4) with very little difference between themselves and the client or with a sentiment of higher importance for the client. 

In question 13 we asked, “Please explain why you perceive the different forms of privacy listed above as being important, or unimportant for your clients.” To give a few quote,

“It is very difficult to make blanket statements about “Client”. In our work we deal with a wide range of people with differing abilities. This range would have highly capable and intellectually astute folks at one end. These people would most likely share a similar concern for privacy as I do. On the other end we have people with low expressive communication and significant intellectual impairment. Through a life of invasive personal care in institutional settings (both group homes and larger facilities) these people have accepted that their privacy is not their own to manage. Although this is unfair and offends my rights based Canadian mentality it is the truth. Similarly, I think we encounter what Stanley Hauerwas refers to as a “failure of imagination” in our dealing with people with intellectual disabilities. The truth is that I cannot imagine how many of our clients perceive themselves or the world around them. Because of this fact I imagine what “I”, with my intact intellectual capacity, would feel like if I found myself having an intellectual impairment. My imagination probably fails to capture how many of our clients actually experience their world and they may very well feel completely differently than I believe that they do.”

**Table 3:** Participant ratings of the importance of various privacy forms to themselves.

Me	Very Important	Important	Moderately Important	Of Little Importance	Unimportant	Response Count
Privacy of Location	**38.9% (14)**	33.3% (12)	11.1% (4)	16.7% (6)	0.0% (0)	36
Privacy of health information	**63.9% (23)**	22.2% (8)	13.9% (5)	0.0% (0)	0.0% (0)	36
Privacy of thought	**63.9% (23)**	27.8% (10)	8.3% (3)	0.0% (0)	0.0% (0)	36
Privacy of memory	**58.3% (21)**	27.8% (10)	8.3% (3)	5.6% (2)	0.0% (0)	36
Privacy of non-health information	**41.7% (15)**	27.8% (10)	25.0% (9)	5.6% (2)	0.0% (0)	36

**Table 4:** Participant ratings of the importance of various privacy forms to their clients.

	Very Important	Important	Moderately Important	Of Little Importance	Unimportant	Response Count
Privacy of Location	**52.8% (19)**	19.4% (7)	16.7% (6)	11.1% (4)	0.0% (0)	36
Privacy of health information	**69.4% (25)**	19.4% (7)	8.3% (3)	2.8% (1)	0.0% (0)	36
Privacy of thought	**66.7% (24)**	19.4% (7)	11.1% (4)	2.8% (1)	0.0% (0)	36
Privacy of memory	**55.6% (20)**	30.6% (11)	11.1% (4)	2.8% (1)	0.0% (0)	36
Privacy of non-health information	**50.0% (18)**	27.8% (10)	13.9% (5)	8.3% (3)	0.0% (0)	36

“There are some things for the clients that really wouldn’t actually affect them if others knew but they deserve to have what they want or where they live or have to be private as we would all be able to choose who we tell what in most situations in our clients cases they need the staff to know and speak for them to who it concerns and nobody else.”

“I think privacy is important to those we support. However many of our participants want us to write things down for them or keep logs/diaries/histories/photoalbums that we or they can refer back to, if they can't remember. Most of our participants recognize that staff change and that if the information is not written down and they themselves are unable to remember - no one will know the information/stories/history *etc*. In cases where guardianship orders exist the guardian often wants us to track everything; medical, social, financial *etc*. and they do not seem as concerned about privacy.”

“All forms are important to my client because they are much more vulnerable than I am.”

In question 14 (Table 5) we asked, “How would you rank your ability to control each of these forms of privacy.”

**Table 5:** Participant rankings of personal ability to control various forms of privacy.

	1 no control	2 minimal control	3 moderate control	4 mostly controlled	5 complete control	Rating Average	Response Count
Privacy of Location	2.8% (1)	**30.6% (11)**	27.8% (10)	**30.6% (11)**	8.3% (3)	3.11	36
Privacy of health information	5.6% (2)	25.0% (9)	16.7% (6)	**36.1% (13)**	16.7% (6)	3.33	36
Privacy of thought	5.6% (2)	0.0% (0)	16.7% (6)	36.1% (13)	**41.7% (15)**	4.08	36
Privacy of memory	5.7% (2)	0.0% (0)	22.9% (8)	34.3% (12)	**37.1% (13)**	3.97	35
Privacy of non-health information	2.8% (1)	**36.1% (13)**	22.2% (8)	30.6% (11)	8.3% (3)	3.06	36

In question 15 (Table 6) we asked, “How would you rank your clients ability to control each of these forms of privacy?”

**Table 6:** Participant rankings of clients ability to control various forms of privacy.

	1 no control	2 minimal control	3 moderate control	4 mostly controlled	5 complete control	N/A	Rating Average	Response Count
Privacy of Location	36.1% (13)	**38.9% (14)**	13.9% (5)	8.3% (3)	2.8% (1)	0.0% (0)	2.03	36
Privacy of health information	**36.1% (13)**	25.0% (9)	19.4% (7)	19.4% (7)	0.0% (0)	0.0% (0)	2.22	36
Privacy of thought	8.3% (3)	16.7% (6)	22.2% (8)	25.0% (9)	**27.8% (10)**	0.0% (0)	3.47	36
Privacy of memory	14.3% (5)	11.4% (4)	25.7% (9)	**28.6% (10)**	20.0% (7)	0.0% (0)	3.29	35
Privacy of non-health information	25.0% (9)	**33.3% (12)**	27.8% (10)	11.1% (4)	2.8% (1)	0.0% (0)	2.33	36

As to control over various types of privacies (question 14 and 15) (Table 5, Table 6), participants felt the most control over their thoughts and memory but only moderate control over privacy of location and medical and non-medical information. In all cases, the staff perceived the control for themselves as higher than the control they perceived their clients as having; perceptions of client control ranged from minimal to moderate throughout. 

To give two quotes,

“Again, given the wide spectrum that I am working with it is difficult to label any of these categories in any accurate way. For some of the clients we serve they have much the same control that I have because they live independently and manage much of their own personal information. For others they have very limited control because they live and work in controlled settings in which their personal information is moderated through staff members.”

“So Many records and documents have been created over the course of our participants lives, and although we know what we collected and how we store it and protect it. As service providers we are obliged to collect this information, thus our participants have little control over the collection of this information. Also because most of our participants have been the recipients of service over the entire course of their life time, many records have been created and collected about them and I am not sure how confidentially these records were kept.”

## 3. Discussion

No current literature addresses perspectives of sensor types or sensor associated privacy issues, for either people with intellectual disabilities or their support staff. We did not find previous studies looking at the perception of community support staff of people with disabilities or at members of disability organizations. As a result, the data generated by our study cannot be compared to any existing knowledge on how staff of disability service organizations in similar employment situations may view the topic investigated in this paper. However, data exists that covers the views of other groups towards different sensors. A qualitative study called “Consumers’ perspectives of wireless cardiac monitoring: Results of a small New Zealand telehealth project” [56], looked at the views of seven New Zealand consumers with a diagnosed heart condition. The study found that the respondents would feel safer if monitored and that the consumer was both “pleased and concerned about seeing their cardiac rhythm” [56]. Consumers mentioned important issues such as skin care to attach the sensor, discreetness of the monitoring equipment and its non-interference with their life as important, as well as training in the use of the equipment and the ability to understand the data from the cardiac monitor. This study acknowledged that the small sample size was a limitation and highlighted that “a larger study is needed, with a wider range of participants that includes the competence factors of literacy, dexterity, vision, hearing, learning ability, memory, training, experience, and language barriers” [56]. Our study looked at staff serving people with learning and other intellectual disabilities. Indeed one extension of our study will be to interview people with intellectual disabilities and parents whereby for people with disabilities we will have to modify the survey into plain language to make it understandable. 

The qualitative study of eight elderly people, age 65–80, “Perceptions of the Elderly on the Use of Wireless Sensor Networks for Health Monitoring” [57] found “a general preference for an embedded sensor implementation *versus* a wearable or ambient implementation, the expressed need of the elderly to have some ability to control / interact with the sensors and the general positive level of support for the idea of sensor-based health monitoring” [57]. The apprehension toward wearable sensors was mainly that in many places like showers one might not wear them and that wearable sensors might not be discrete enough [57]. We did not ask our respondents explicit whether they and their clients would prefer wearable over implantable or ambient sensors but given that many respondents believed that many of the clients would not be able to work with a sensor we submit that the implantable or ambient one might be seen as preferable over a wearable one in the case where they believe the client would be unable to deal with the wearable sensor. On the other hand, the wearable sensor option might be seen as preferable over the implantable one due to its reversibility an option many respondents mentioned as important and over ambient option given that privacy was seen as so important and that the respondents felt that their clients already had lack of control over their privacy.

A 2006 online open question survey with 103 respondents from Europe, Australia, Canada and the United States, “Exploring the Benefits of Using Motes to Monitor Health: An Acceptance Survey” [35] found the main concerns being security, privacy and trust. Trust and privacy was also seen as important in the study “Adult Children’s Perceptions of Intelligent Home Systems in the Care of Elderly Parents” [58]. This fits on the one hand with the views of our respondent who saw privacy to be a main issue, however our respondents did not mention security and mentioned trust only once in context of the importance of the relationship between staff and clients. Given that some respondents felt that some of their clients would not understand the sensors, they might not be able to trust, as trust can be linked to understanding. 

The qualitative study of eight elderly age 65–80, “Using Wireless Sensor Networks for Aged Care: The Patient's Perspective” [59] found that elderly acceptance of sensor technology are linked to cost and control. This is in sync with our findings that also saw cost and control as main issues. The qualitative study of 15 elderly people “Older adults’ attitudes towards and perceptions of ‘smart home’ technologies: a pilot study”, highlights the concerns around “user-friendliness of the devices, lack of human response and the need for training tailored to older learners” [60]. The issues of user-friendliness as well lack of human response were main concerns of the respondents in our study. 

The study, “Disability, Age, and Informational Privacy Attitudes in Quality of Life Technology Applications: Results from a National Web Survey [61] of n = 1,518 respondent looked at “perceived acceptability of sharing information about toileting, taking medications, moving about the home, cognitive ability, driving behavior, and vital signs with five targets: family, healthcare providers, insurance companies, researchers, and government” [61]. They looked further at “acceptability of recording the behaviors using three methods: video with sound, video without sound, and sensors”. Sharing or recording of information about toileting behavior was the least accepted one, as was the sharing of information with the government and insurance companies. Recording with sensors was seen as more acceptable than using video with or without sound [61]. The study found that respondents identifying as disabled people were more accepting of sharing and recording of information than nondisabled adults. They stated that people with disabilities that needed help with taking medication, preparing meals, doing laundry, cleaning, or shopping) were more accepting than non-disabled respondent and less accepting than disabled people that also needed help with bathing, dressing, grooming, eating, or transferring [61]. This finding indicates that disabled people are not a homogenous group and that much more research is needed that ascertains the views of disabled people with various ability differences including intellectual differences. 

One study [44] found that older adults are willing to trade privacy for autonomy. We did not give our respondents specific trade-offs to think about. The trade-off between privacy *versus* autonomy was not mentioned in our study. This might be due to staff seeing themself as autonomous and their clients as lacking privacy in so many areas. However, if we had posed this question specifically we might have seen the trade-off appearing. This is a question that should also be asked of people with intellectual and other disabilities as it cannot be expected that all disabled people respond in the same way as [61] showed. Another study found trade-off between privacy and utility [62] something we also found with our respondents.

No surveys exist in regards to many of the topics covered herein, such as the issues of privacy of thought, privacy of memory and the use of neurotransmitter sensors. In general existing survey data and our data suggest that there is a need for more surveys covering more aspects of sensors as well of other groups that will be impacted by advancements in sensor technologies. 

## 4. Experimental

An online delivered exploratory non-probability non generalizable survey (using a combination of 55 simple yes or no, Likert scale, as well as opinion rating scale questions) was developed. The executive director of the disability service organization with whom we discussed the project before we developed the survey saw a draft of the full survey and commented on language and clarity. We made adjustments in accordance to the executive director suggestions and the final version was forwarded for ethics approval. Various questions had the options of giving comments. Given the discussion with the executive director it was anticipated that staff would also give comments related to questions that had a comment box. The survey received ethics approval from the University of Calgary Health Research Ethics board. The executive director gave the link to the online survey to the staff of the organization after we received ethics approval. Quantitative and qualitative data presented here were generated through questions 12–15 on the topic of privacy, questions 16–19 on the topic of sensors and questions 20–22 on the topic of the quantified-self movement using the online Survey Monkey Platform. Six questions covered various demographic angles. All 44 survey recipients answered at least 1 content question, reflecting a response rate of 100%. In accordance with the ethics approval, all answers were voluntary with participant withdrawal possible at any time. The response rate per question covered in this paper was very high and ranged between 64% and 73%. As a result, important insight into what staff of this one disability service organization thinks in regards to the topic was obtained. Although the results cannot be generalized to the disability service industry as a whole, we submit the generated data and the survey might be useful as a first step in generating more empirical data from staff of disability service organizations allowing for the comparison of the views of staff of different disability service organizations. Data of this survey were seen to provide an avenue for staff of disability service organizations to voice their opinions on upcoming health technology discourses that they are not normally present within. Quantitative data was extracted using survey monkey intrinsic frequency distribution analysis capability. The data was exported as pdf file into Atlas-ti for the qualitative analysis of the comment box contributions. 

## 5. Conclusions

Empirical data of the views of staff of disability service organizations that work within the community rehabilitation, rather than the clinical setting in regards to emerging health technologies, are rare. Our results indicate that in general, staff do not have a negative view of sensors but a differentiated one that is more skeptical towards certain sensor applications than others. Furthermore, although staff cherishes privacy for themself and their client, they feel that they have only moderate control over their own privacy and they feel that their clients have even less control. These results suggest that some work still has to be done in this area and that privacy may be a barrier for the adoption rate of the different sensor types covered in this study. The views of staff that their clients very likely will not be able to be an active user of the technology and will only be able to be observed by others who control the sensor and the data, indicates an area of needed improvement for how sensors of various applications are deployed and developed. We submit that these views highlight challenges to come for people with intellectual disabilities and their support network; if a more active role is expected of health consumers in the future, the disabled person has to fulfill this role. If the disabled person cannot fulfill the role due to intellectual, physical or other type of inaccessibility of the products, their support network will have to fulfill this role for them. Numerous endeavors are underway to increase health literacy in light of the shift towards individuals as active shapers of their health and health care decisions—however, few efforts are evident concerning universal design (which includes intellectual accessibility) of health technologies or to increase health literacy of people with intellectual disability. We submit people with intellectual disabilities and their networks, such as parents and non-family support staff, have to be involved in sensor as well as quantified-self discourses to ensure that this group is not put at an undue disadvantage. The staff interviewed in our study felt that their clients already experienced a lack of privacy and minimal control over their privacy; we see this as a problematic message requiring acknowledgement, especially in sensor discourses, particularly in countries that cherish privacy. We also submit, given that existing surveys show various trade-offs, that another reason to increase stakeholder involvement is the finding that staff felt that their clients would not be able to use most wearable sensors in development. Given that surveys must modify language and terminology used in order to ascertain sensor acceptability—due to intellectual ability differences of the target group—what we suggest here extends beyond the delivery of existing surveys to consumers with intellectual disabilities; new plain language has to be developed that allows people with intellectual disabilities to understand the issue and to provide their input. 
